# Enhanced Methylation Analysis by Recovery of Unsequenceable Fragments

**DOI:** 10.1371/journal.pone.0152322

**Published:** 2016-03-31

**Authors:** Gordon R. McInroy, Dario Beraldi, Eun-Ang Raiber, Katarzyna Modrzynska, Pieter van Delft, Oliver Billker, Shankar Balasubramanian

**Affiliations:** 1 Department of Chemistry, University of Cambridge, Cambridge, Cambridgeshire, United Kingdom; 2 Cancer Research UK Cambridge Institute, Li Ka Shing Centre, Cambridge, Cambridgeshire, United Kingdom; 3 Wellcome Trust Sanger Institute, Cambridge, Cambridgeshire, United Kingdom; 4 School of Clinical Medicine, University of Cambridge, Cambridge, Cambridgeshire, United Kingdom; CEA - Institut de Genomique, FRANCE

## Abstract

Bisulfite sequencing is a valuable tool for mapping the position of 5-methylcytosine in the genome at single base resolution. However, the associated chemical treatment causes strand scission, which depletes the number of sequenceable DNA fragments in a library and thus necessitates PCR amplification. The AT-rich nature of the library generated from bisulfite treatment adversely affects this amplification, resulting in the introduction of major biases that can confound methylation analysis. Here, we report a method that enables more accurate methylation analysis, by rebuilding bisulfite-damaged components of a DNA library. This recovery after bisulfite treatment (ReBuilT) approach enables PCR-free bisulfite sequencing from low nanogram quantities of genomic DNA. We apply the ReBuilT method for the first whole methylome analysis of the highly AT-rich genome of *Plasmodium berghei*. Side-by-side comparison to a commercial protocol involving amplification demonstrates a substantial improvement in uniformity of coverage and reduction of sequence context bias. Our method will be widely applicable for quantitative methylation analysis, even for technically challenging genomes, and where limited sample DNA is available.

## Introduction

5-methylcytosine (5mC) is the primary epigenetic DNA modification in eukaryotes. This covalent base modification regulates gene expression and is important to genomic imprinting [1] and disease states across a wide range of organisms [2, 3]. Accurate, quantitative detection and mapping of 5mC in genomic DNA is essential to understand its function. The core methodology used to provide single base resolution methylation maps is bisulfite sequencing (BS-seq) [4], which exploits the differential deamination kinetics of cytosine and 5mC when treated with sodium bisulfite [5]. Bisulfite treatment causes cytosine to rapidly deaminate to uracil, while 5mC reacts over two orders of magnitude slower. Subsequent sequencing reveals cytosine to thymine switches at unmodified cytosine sites and cytosine calls at 5mC loci. Additionally, BS-seq may be used to measure the extent of methylation at a single genomic locus in a population of cells, by dividing the number of reads carrying an unconverted cytosine by the total number of reads covering that site. For genomes containing 5-hydroxymethylcytosine (5hmC) [6, 7] it is essential to be aware that 5mC and 5hmC are indistinguishable in BS-seq data [8]. New methods have been developed to distinguish 5mC from 5hmC [9, 10].

While BS-seq has been regarded as the gold standard for methylation analysis, there are some serious deficiencies with the method. One major issue is the formation of abasic sites via the loss of pyrimidine bases [11]. Heat or alkali conditions can induce strand scission at these sites, both conditions which are employed during bisulfite treatment. Current library preparation techniques require addition of sequencing adapters to both ends of the fragmented DNA of interest prior to bisulfite conversion [12, 13]. The retention of these ligated adapter sequences at both ends of a fragment is an absolute requirement for generation of a read during sequencing; consequently just one DNA cleavage event precludes data acquisition. As a result of the need to ensure complete cytosine conversion, harsh bisulfite conditions can induce strand scission that renders up to 99.9% of DNA fragments in a library unsequenceable [11]. PCR amplification is therefore required to enrich for the remaining minority of uncleaved fragments bearing both adapter sequences. Bisulfite induced strand scission has been exploited to fragment genomic DNA in the post-bisulfite adapter tagging method [14]. This method has enabled notable advances such as single-cell genome-wide bisulfite sequencing [15].

Post-bisulfite DNA is invariable AT-rich due to the conversion of cytosine to uracil. Even originally balanced genomes become highly skewed, for example the AT-content of a human genome rises from approximately 57% to 78% following bisulfite conversion. Sequences with highly skewed base compositions amplify poorly or not at all [16], and therefore require an increased number of PCR cycles to obtain sufficient material for sequencing. Moreover, the ratio of DNA fragments following amplification is not truly representative of the input material, as those fragments tending towards a more balanced AT/GC composition will be amplified preferentially. Therefore, the accuracy of ‘quantitative’ methylation data, which has been generated from PCR amplified DNA libraries, must be drawn into question. Fragments of DNA containing 5mC will retain a more balanced AT/GC composition than those fragments without the modification, due to the retention of 5mC and conversion of cytosine to uracil. Amplification can lead to the overrepresentation of DNA fragments with a more balanced composition, and thus an overestimation of methylation levels [17].

Previous studies have shown that prudent enzyme choice and minimizing amplification cycles can limit enrichment biases in bisulfite data [17], though not completely evade them. One method designed to produce representative sequencing libraries from samples requiring amplification is the linear amplification for deep sequencing (LADS) protocol [18]. This method relies upon *in vitro* transcription for amplification before cDNA synthesis to regenerate a sequenceable library, and has only slightly decreased coverage uniformity compared to amplification free techniques. However, it has not yet been applied to bisulfite treated genomic samples for methylation analysis.

We have developed a PCR-free library preparation for bisulfite sequencing. A two-step ligation protocol enables us to rebuild ‘damaged’ fragments into sequenceable strands, thus regaining library diversity and quantity. As a result, we obtain virtually unbiased data from low nanogram quantities of input sample. We employed our method to obtain the first methylome of the blood-borne stages of the murine malarial model *Plasmodium berghei*, which has a starting genome composition of 78% AT and so poses a challenge for amplification dependent techniques. While epigenetic control mechanisms in *Plasmodium spp.* have attracted much study [19–21], DNA modification has been largely neglected. The ability to obtain an accurate methylation map would add to the knowledge base of the existing epigenetic network, and may offer new therapeutic targets.

## Results

### 0.1 Preparation of PCR-free bisulfite libraries

Whilst bisulfite treatment depletes sequenceable DNA due to loss of adapters by fragmentation, the majority of cleaved fragments still contain useful information and are of a mappable length. We recover these lost fragments and the associated information by employing a two-step ligation procedure, where the P7′ adapter is added before bisulfite treatment and the P5 adapter afterwards.

The recovery after bisulfite treatment (ReBuilT) method begins with fragmentation, end repair and A-tailing. We then employ custom methylated adapters, with one strand bearing a 3′ biotin label and the other a 3′ dideoxythymidine (ddT) terminator. The presence of a 3′ ddT prevents ligation to the 5′ end of the insert DNA, resulting in a single-stranded directional ligation to the 3′ insert terminus. Furthermore, adapter dimerisation is not possible during this ligation, thus preventing formation of common sequencing contaminants. Following bisulfite conversion, primer extension with a high fidelity uracil tolerant polymerase generates blunt ended double stranded DNA, which is immobilized on streptavidin coated magnetic beads via the biotin label. Immobilization enables near lossless manipulation of the library during subsequent processes. The immobilized DNA is A-tailed before ligation of a complementary P5 adapter. The biotin bearing strand of this fully adapted DNA contains uracils, so is not suitable for standard next-generation sequencing, while the other strand contains only the canonical nucleobases. Denaturing conditions elute the canonical DNA strand ready for sequencing.

As a proof of concept experiment we generated sequencing libraries from *E. coli*, chosen due to its small genome (4.6 Mb) and balanced base composition (50%). We employed qPCR to compare the concentration of sequenceable fragments obtained with either ReBuilT or a standard BS-seq library preparation protocol. With equal input quantities of DNA, the concentration of sequenceable fragments was two orders of magnitude higher with the ReBuilT protocol than with the standard protocol excluding PCR amplification (S1 Fig). We then sequenced the libraries, having amplified the BS-seq library to obtain sufficient adapter ligated DNA for sequencing, with paired end reads on an Illumina MiSeq. Upon inspection of the genomic coverage, the ReBuilT method had a significantly more uniform profile than the amplified library (termed PCR-BS). Notably, a number of regions had very few reads in the PCR-BS dataset, yet were efficiently sequenced via the ReBuilT method (S2 Fig).

With this promising method in hand, we focused on our system of interest, the challenging AT-rich *P. berghei* genome. Since there have been no reports of the DNA base composition of *P. berghei*, we first analysed the global DNA modification levels by tandem mass-spectrometry. We found the level of 5mC to be 0.31% of total cytosine species, and detected no other oxidised cytosine derivatives (S3 Fig).

We employed the ReBuilT method to generate PCR-free libraries from 50 ng of *P. berghei* DNA, extracted from an asynchronous population of erythrocytic stages. In parallel we generated traditional bisulfite libraries that included post-bisulfite PCR amplification (again termed PCR-BS). We sequenced multiplexed libraries on the Illumina NextSeq platform, with paired end reads of 75 or 100 bases. We obtained up to 285 million reads from 13% of an amplification free library generated from 50 ng, i.e: equivalent to 6.5 ng of input DNA, which provided ample data for analysis of low methylation levels with high confidence.

### 0.2 Comparison of sequencing data quality

**Fig 1:**
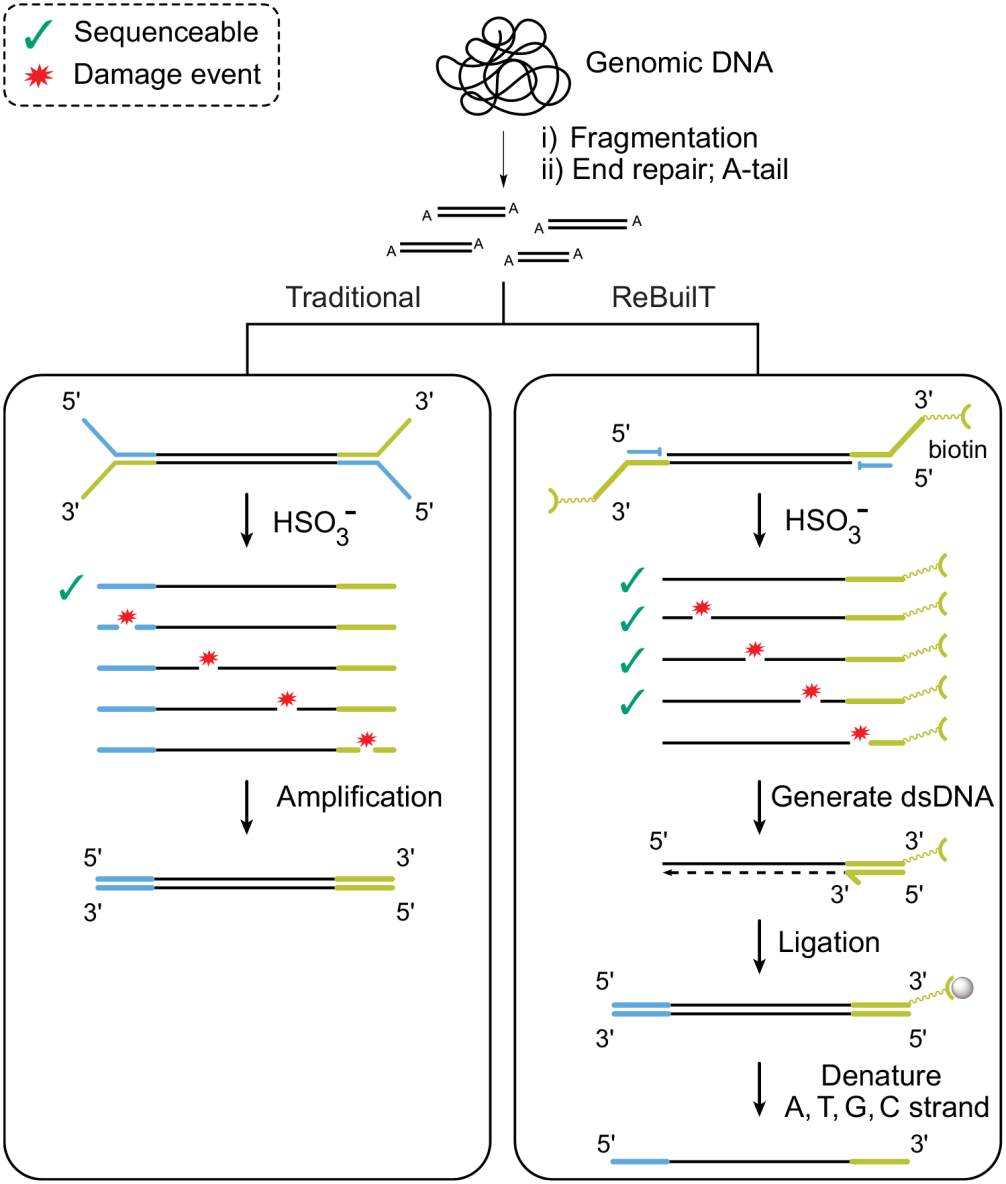
Schematic describing key differences between the ReBuilT and PCR-BS protocols. The possible fragments generated by cleavage (indicated with red stars) during bisulfite treatment are illustrated, and annotated to indicate if they remain sequenceable. **Left track:** adapter ligation precedes bisulfite treatment, after which the few surviving fragments are amplified by PCR. **Right track:** a single-stranded 3′ selective adapter ligation precedes bisulfite treatment. A primer extension generates dsDNA, which is immobilized on magnetic beads. A second ligation is performed before the non-uracil containing strand is denatured ready for sequencing.

To evaluate the possible benefits of ReBuilT over the PCR-BS method, we compared a range of data quality metrics for the two systems (Fig 1). As the sets of libraries were generated from the same source of genomic DNA, any differences should be solely due to the library preparation method.

#### Retention of raw data

**Fig 2:**
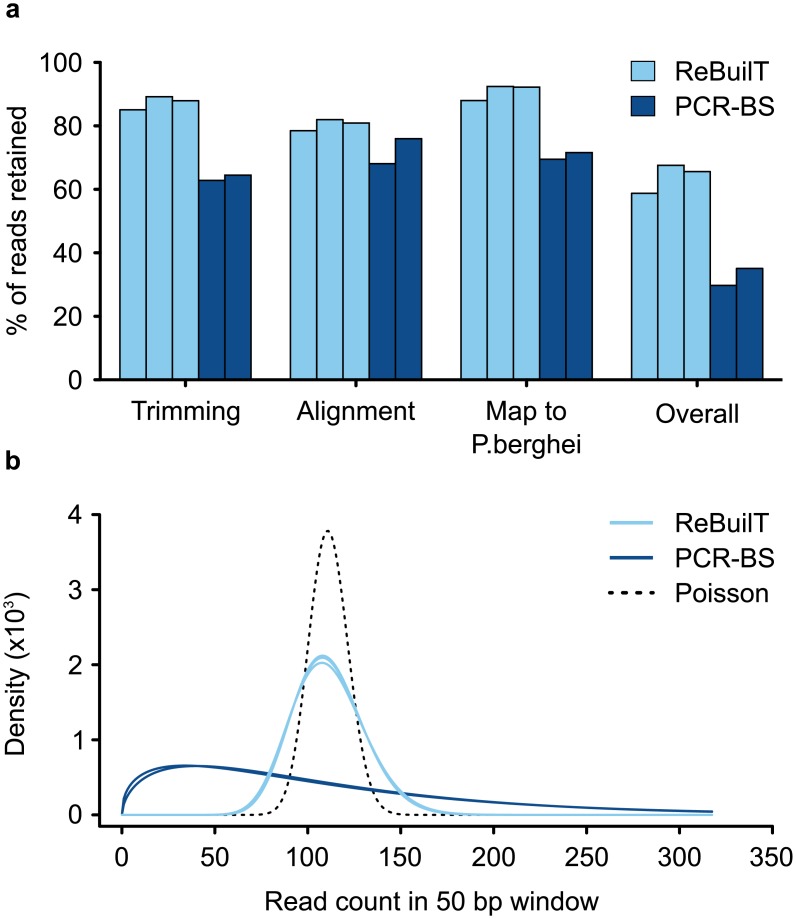
ReBuilT produces superior quality sequence data. (a) Retention of raw data through bioinformatic preprocessing. Trimming indicates removal of adapter or low-quality sequences; alignment was to a chimeric *P. berghei*/*M. musculus* genome to remove host contamination; Map to *P. berghei* shows the fraction of reads aligning to the parasitic genome. ReBuilT retained almost twice percentage of raw data. (b) The distribution of mapped reads in 50 bp windows across the genome. The Poisson distribution (dashed line) describes the ideal distribution in the absence of external biases. REBUiLT approximates this distribution, while PCR-BS does not.

We first looked at how much raw sequencing data was retained following a bioinformatic pipeline including adapter trimming, quality trimming and read alignment (Fig 2a). Following all trimming steps the ReBuilT libraries retained on average 87.4% of the raw data, compared to 63.6% for the PCR-BS libraries. The majority of the PCR-BS loss was due to removal of dimeric adapter sequences, which cannot form in the initial ReBuilT ligation and are not retained during the second ligation. Surviving reads were aligned to a chimeric *P. berghei* and *M. musculus* reference genome, as extracted parasite DNA may be contaminated with some genomic material from the host. Average alignment rates to this chimeric reference were 80.5% and 72.1% for the ReBuilT and PCR-BS samples respectively. From this subset of aligned reads 90.9% of ReBuilT and 70.6% of PCR-BS reads were aligned to the *P. berghei* reference genome, with the remaining reads aligning to the host mouse genome. Following all data processing, 58.8% of the raw ReBuilT data could be used for methylation calling, while the PCR-BS libraries retained only 29.7% of the raw data. The ReBuilT method, therefore, yields considerably more useable data, which reduces the sequencing power required for methylation analysis.

#### Sequenced insert size

The sequenced insert size in ReBuilT libraries was expected to decrease from the initial 250 base pairs, due to the recovery of short fragments damaged during bisulfite treatment. Indeed, we found the mean insert length to be 111bp with a mode of 60bp (S4 Fig). Surprisingly, the PCR-BS insert size also decreased to a mean of 108 and a mode of 80. We expect this shift is due to the survival rate of a fragment through bisulfite treatment being inversely proportional to its length. Notably, the number of reads in the 200–300 bp range was higher with ReBuilT (8.1%) than PCR-BS (4.4%). Above a certain length the chance of a fragment not suffering a single bisulfite-induced scission event becomes very small, so long fragments are depleted from the PCR-BS libraries. However, the ReBuilT method is capable of recovering these long strand fragmentation products.

#### Uniformity of sequencing coverage

For optimal whole genome bisulfite analysis it is essential that the read depth remains even across the genome. Regions with uneven coverage can otherwise exhibit inaccuracies in apparent methylation levels. To address this issue, we down-sampled libraries to be of equal size, and examined the read depth distribution (S5 Fig). The ReBuilT libraries consistently exhibit a higher normalised median read depth, and a dramatically reduced standard deviation, than the PCR-BS libraries (ReBuilT: 2.7 ± 1.7; PCR-BS: 1.9 ± 5.1). We further addressed this point by plotting the data as a density histogram, and overlaying the Poisson distribution expected in the complete absence of bias (Fig 2b). While the ReBuilT libraries approximate the expected distribution, the bias introduced by PCR amplification is quite striking. The PCR-BS data is heavily skewed to low read counts, and has a tail stretching towards very high values. This result can be interpreted as the majority of regions experiencing inadequate coverage, while a small subset of regions are significantly over represented at their expense. In such regions with low coverage, there is a reduced ability to confidently detect methylation levels.

#### Duplication rate

When sequencing a small genome, a certain proportion of apparent duplicates are inevitable, due to a limited range of positions at which fragments can possibly start and end. For the number of reads obtained, the expected duplication rate is approximately 12%. The ReBuilT libraries were found to have an average duplication rate of 16% (S6 Fig). Clearly there are no PCR duplicates, as no amplification has been performed; however, this increase is a reflection of the imperfect overlap with the Poisson distribution seen in Fig 2b. The PCR-BS sample has a duplication rate almost double at 30%, which is a cumulative effect of amplification duplicates and extremely uneven coverage. Uneven coverage leads to peaks and troughs in read depth, which will locally raise or lower the expected duplication rate.

#### Sequence composition bias

**Fig 3:**
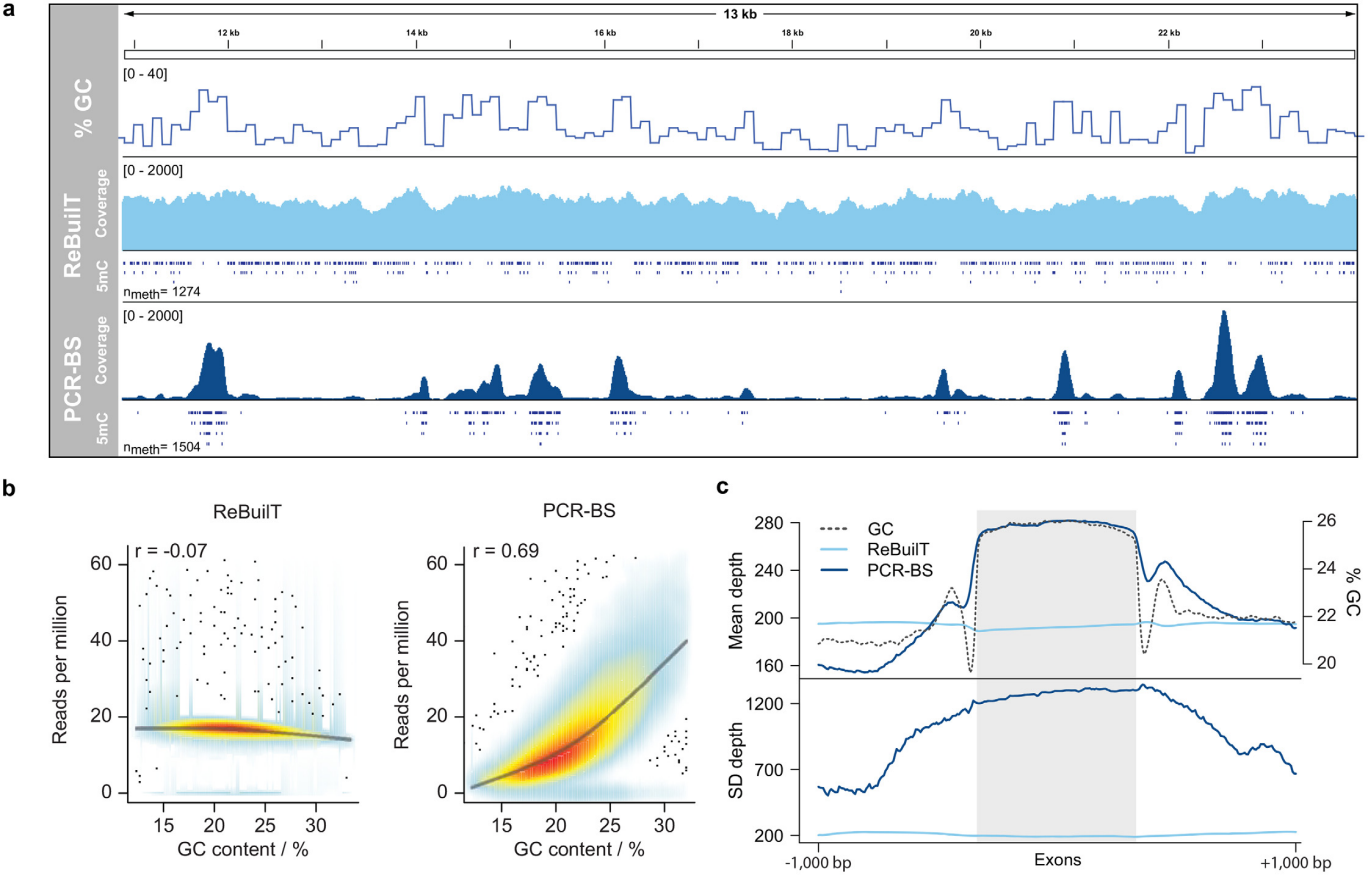
Effect of GC content on uniformity of coverage. (a) Genome browser view showing the coverage obtained across a region from *P. berghei* for both methods. While near constant for ReBuilT, distinct read pile-ups in PCR-BS that appear to track GC content. (b) The GC content was calculated in 300 bp windows and plotted against the normalized informative read count. PCR amplification induces a strong preference for more balanced base compositions. (c) In *P. berghei* the GC content has a distinct profile across exons (dashed line). ReBuilT coverage is unaffected across this genomic feature, while PCR-BS tracks the GC percentage closely.

In Fig 3 we show the dependency of read count on the local GC content of the reference genome. Data for individual libraries is shown in S7 Fig. The ReBuilT data shows little sensitivity towards GC content, even with the extremely skewed composition of the *P. berghei* genome, as shown by the near horizontal smoothed regression line. In stark contrast, the PCR-BS data exhibits a clear preference for more GC-rich windows. A clear example of this effect can be seen in Fig 3a, which shows the level of coverage at each base within a region, and the GC content in 100 base windows. While the read count is evenly spread in the ReBuilT track, the PCR-BS reads are more likely to fall in regions of high local GC content. The confidence of methylation calls is reduced in areas poorly covered, as can be seen by the lack of methylation in low GC regions for PCR-BS.

This amplification induced GC bias explains the difference in alignment rates to the mouse genome seen in Fig 2a. Despite extensive purification procedures the *P. berghei* DNA sample exhibits minor mouse cell contamination, resulting in the presence of some murine sequences in the data. Although input was obtained from the same sample of purified DNA, the two methods gave disparate levels of mouse contamination. The ReBuilT libraries averaged 9.1% of reads aligning to the mouse genome, while the PCR-BS samples averaged 29.5%. As the *M. musculus* genome is 42% GC and the *P. berghei* genome is 22% GC, we suggest this disparity arises from the preference for amplifying DNA fragments with more balanced sequence compositions.

Certain biological features display characteristic base compositions. Fig 3c shows the average GC content profile across exons in *P. berghei*. The read count profile across the same regions is uniform for ReBuilT data, as expected from Fig 3b. However, the PCR-BS read count closely tracks the GC content, rising over exons and falling in intergenic regions. This bias may lead to an overrepresentation of coding regions in data generation from protocols involving amplification.

Taken together, the above analyses suggest that traditional PCR-dependent bisulfite experiments have poor quantitative power, and may fail to capture methylation sites in certain genomic features. Methods capable of evading PCR amplification can produce markedly improved data that can have a pronounced effect on methylation analysis. The ReBuilT method provides a convenient path to generating sequencing libraries without amplification, even if limited quantities of DNA are available.

### 0.3 Methylation in the *P. berghei* genome

Using the ReBuilT data we found 76,205 methylated loci (FDR corrected p-value *p* < 0.01), representing 1.87% of the total genomic cytosine sites. The global level of unconverted cytosines was 0.70%, with methylation at single sites reaching a maximum of 21%. This low global value conforms to the 5mC we detected by LC-MS/MS (0.33% 5mC/total C). We were able to confidently quantify such low levels of methylation due to the high depth of sequencing we obtained: a combined 600x depth across replicates. The number of sites and the global methylation level detected (5.91%) was substantially higher in the PCR-BS dataset, which also displayed a clear correlation between the percent methylation and read count (S8 Fig).

**Fig 4:**
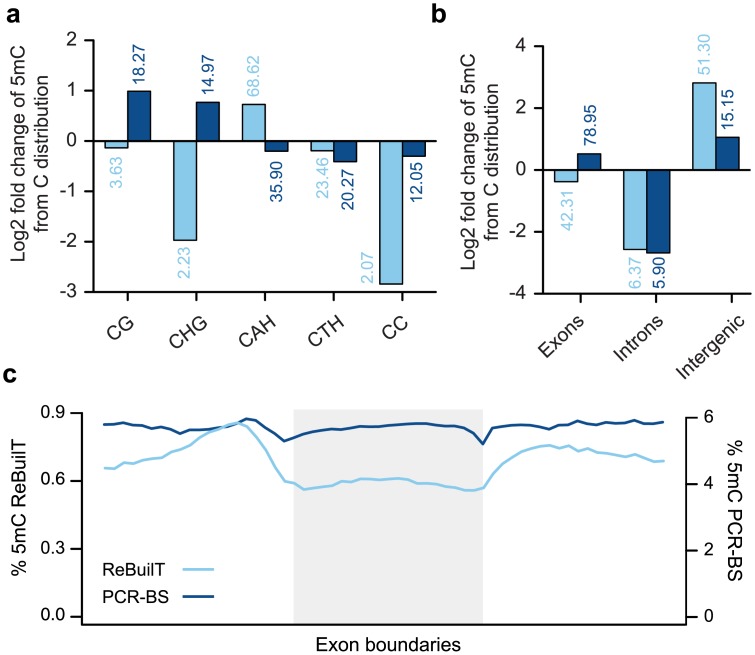
Cytosine methylation in *P. berghei*. (a) The log2 fold change of 5mC contexts from cytosine contexts within the *P. berghei* genome. Each bar is annotated with the percentage of 5mC loci within the context. The sequence context varies greatly between PCR-free and amplified samples. ReBuilT data reveals an enrichment in the asymmetric CAH context, and CHG and CC contexts are strongly disfavoured. PCR-BS methylation occurs more often in CG and CHG contexts (H = A, T, G). (b) The distribution of methylation across genomic regions, shown as log2 fold change from the distribution of cytosines. The percentage of 5mC loci in the regions is given above each bar. Methylation in the AT-rich intergenic regions is underrepresented in the PCR-BS dataset. (c) The profile of 5mC levels over exons. Traditional PCR-BS gives a similar profile to ReBuilT, but greatly over-estimates the 5mC levels.

From the ReBuilT dataset we found the context of methylated loci to be primarily CAH (68.62%) and CTH (23.46%), with the remaining sites being found in CG (3.6%), CHG (2.2%), and CC (2.1%) contexts. While the genomic context of all cytosines shows a preference for adenine in the +1 position, this preference is significantly increased for methylated loci. Fig 4a demonstrates this point by showing the log2 fold change of methylated loci context from the genomic cytosines. All contexts are underrepresented against the *P. berghei* background with the exception of CAH, which is overrepresented. This asymmetric context contrasts with the strong preference for CG methylation in mammalian genomes. Furthermore, cytosine and guanine bases are generally depleted around methylated loci, in agreement with previously reported data from *P. falciparum*[22]. While the majority of methylation from the PCR-BS dataset also occurs within the CAH context, a defining feature is a preference for nearby guanines. This context distribution may be affected by the read count bias towards GC rich regions, such as the exons described in Fig 3c.

The genomic location of methylated loci is shown in Fig 4b. We found the majority of methylated loci (51%) were located in intergenic regions. Methylation within gene bodies was predominantly exonic (42% of all loci, 87% of genic loci); however, as exons make up 55% of the plasmodium genome, and are relatively cytosine rich elements, methylation is slightly underrepresented in these regions. This is confirmed when visualising the methylation profile in and around exons genome-wide (Fig 4c). Interestingly, there is a change in methylation levels across intron-exon boundaries, which by analogy to mammalian systems could be involved in transcript splicing [23]. The discrepancy between ReBuilT and PCR-BS methylation in exonic and intergenic regions may again be due to the GC bias described in Fig 3b & 3c. Intergenic regions may be poorly covered due to their high AT content, reducing detection power, while exonic regions are overrepresented due to preferential amplification, especially of fragments containing unconverted cytosine species.

To identify regions of increased methylation density we applied a hidden Markov model to the significance levels of individual sites. In this way we found 20,823 regions that contained six or more cytosines enriched in methylation, with average methylation levels approximately ten-fold higher than the global value. An example of such a methylation cluster is given in S9 Fig. This non-random distribution of cytosine methylation is a strong indicator of biological significance [24, 25]. Indeed, clusters of methylation are more important than single highly methylated sites for gene silencing [26].

### 0.4 Application to mammalian genomes

To benchmark our method for application towards mammalian genomes, we prepared both PCR-BS and ReBuilT sequencing libraries from E14 mouse embryonic stem cells, and compared the detected methylation levels. We sequenced 351,944,111 read pairs from the PCR-BS library and 160,799,026 read pairs from the ReBuilT library resulting in per-base read coverage of approximately 15x and 5x respectively. The C to T conversion (i.e. the estimate of methylation) was largely consistent between the two protocols, though the ReBuilT library tends to give slightly lower estimates as can be seen in S10 Fig. To explore this comparison further, we looked at the percentage methylation within different contexts (S5 Table). Non-CpG methylation was comparable for the two methods, indicating the bisulfite treatment was efficient in both cases, while methylation in CpG contexts was higher in the PCR-BS than ReBuilT samples (67.84% vs. 60.10%). The overall similarity between the methods shows the ReBuilT method is indeed accurately detecting methylation. Furthermore, S10 Fig demonstrates that amplification biases of bisulfite-converted genomes are not restricted to highly skewed genomes such as *P. berghei*—but affects the commonly studied, balanced genome of *M. musculus*.

## Discussion

The data generated from the ReBuilT method provides compelling evidence for the key benefits of PCR-free methylation analysis. We demonstrate that this approach results in increased uniformity of coverage, a lower duplication rate and substantially reduced sequence context biases as compared to a BS-seq approach requiring PCR amplification. Consequently, the methylation data more accurately represent the true methylome of the organism. Additionally, our findings suggest that standard sequencing of *Plasmodium spp.* would benefit from being performed without amplification, due to the high variation of GC content in certain genomic regions leading to their overrepresentation.

By employing our method in conjunction with high depth next-generation sequencing, we have confidently quantified low levels of methylation in the *P. berghei* genome. Furthermore, the methylation data was generated from only 50 ng of genomic DNA. We found global methylation levels were low, and occurred predominantly in the asymmetric CAH context. This methylation profile is similar to those seen when non-CG methylation is studied in other eukaryotes. As the vast majority of methylation occurs in the asymmetric CAH context, *de novo* methylation must be the primary mechanism of installation. Furthermore, given the low modification levels, it would appear that methylation is not highly conserved between the erythrocytic stages. It is therefore plausible that methylation is used to mark key genes that are required to be active or silent at key points within each life stage. Alternatively it is possible that cytosine methylation is present solely within one of the four life stages present in the mixed erythrocytic population. If this were the case for one of the minor constituents, such as gametocytes, methylation levels would be transiently and significantly elevated. With this new method in hand it will be possible to interrogate purified samples of the distinct life stages within the erythrocytic cycle and resolve this question.

While we have primarily demonstrated the utility of our method on the AT rich genome of *P. berghei*, it has broad applicability and scalability. In this manuscript we have sequenced both highly AT rich genomes (*P. berghei*, 22% GC), and those with more balanced base compositions (*E. coli*, 51% GC). Larger mammalian genomes are also tractable with the ReBuilT approach, with an expected 70-fold coverage obtainable from 50 ng of genomic DNA.

In conclusion, our approach enables the study of methylation in genomes previously intractable to BS-seq, as exemplified by the malarial parasite *P. berghei*. Our findings suggest that prior analysis based on PCR-BS may be subject to inaccuracies and misinterpretation. Genomic regions that have very high AT%, such as greater than 80%, following bisulfite treatment may have been missing from previous data sets or assigned incorrect methylation levels. The amplification biases we have described suggest careful consideration should be given for the interpretation of data obtained from bisulfite approaches involving PCR amplification.

## Materials and Methods

### Ethics statement

All animal experiments were conducted under a project license from the UK Home Office in accordance with national and European animal welfare guidelines.

### *P. berghei* culture and DNA extraction

6–10 week old Theiler’s Original (TO) mice were injected intraperitoneally with 0.2 mL of 6 mg/mL phenylhydrazine, and three days later infected with 5 × 10^7^ parasites (*Plasmodium berghei* ANKA strain, clone 2.33). From day six onwards tail smears were taken to assay parasitaemia. Mice were bled by cardiac puncture and parasitic DNA extracted following standard protocols [27].

### Sonication of genomic DNA

500 ng DNA (10 mM tris-HCl pH 8, 1 mM EDTA) was sheared by sonication with the Covaris M220 focused-ultrasonicator to give an average fragment length of 250 base pairs (peak incident power 50W, duty factor 20%, 200 cycles per burst, 120s treatment time). The amount of DNA was quantified with the Qubit dsDNA broad range assay and fragmentation confirmed with the Agilent 2200 Tapestation using D1000 screentapes and reagents.

### DNA digestion and LC-MS/MS analysis

250 ng genomic DNA was digested with DNA degradase (Zymo research) following the manufacturer’s instructions, with stable isotope labelled nucleotides (dC + 3, m5C + 3 and hm5dC + 3) spiked in at 25 nM final concentration. A dilution series (0.0125–15000 nM) of the unlabelled reference standards (dC, 5mC and 5hmC; Sigma Aldrich, Carbosynth Ltd) were also mixed with the stable isotope labelled nucleosides.

Quantitative LC-MS/MS analysis was carried out using an Agilent 1290 Infinity UHPLC coupled to a Thermo Q-exactive mass spectrometer. LC was performed on a Waters Acquity UPLC HSS T3 column (100 x 2.1 mm, 1.8 μm particle size) kept at 50, applying a gradient starting at 100% of 0.1% formic acid in water followed by increasing proportions of 0.1% formic acid in acetonitrile up to 30%, at a flow rate of 350 μL/min over 3 minutes. The MS was operated using HESI in positive ion mode, with spray voltage of 4 KV, heater temperature of 350°C and capillary temperature of 320°C. The instrument was present to isolate the precursor ions for dC (258 m/z), dC+3 (321 m/z), 5mC (242 m/z), 5mC+3 (245 m/z), 5hmC (258 m/z) and 5hmC+3 (261 m/z).

### Generating modified adapters

Sequences given in S6 Table. ODN1a was purchased (ChemGenes) or generated by end-labelling the (n-1) sequence with dideoxythymidine triphosphate (TriLink) using terminal deoxynucleotidyl transferase (NEB). All cytosines in ODN1b were replaced with 5mC to retain the adapter sequence following bisulfite conversion. Adapter pairs were annealed in a thermocycler (95°C for 10 minutes, cooling to 70°C over 10 minutes, holding at 70°C for 10 minutes and then slowly cooling to room temperature at 0.1°Cs^-1^) to give 25 μM solutions in 10 mM Tris-HCl pH 7.4, 50 mM NaCl. Annealing ODN1a and ODN1b provided adapter pair 1; annealing ODN2a and ODN2b provided adapter pair 2.

### ReBuilT protocol

A detailed protocol is provided as supplementary material, S1 Protocol. In brief, 50 ng of sonicated genomic DNA was blunt ended, phosphorylated and dA-tailed, before ligation of custom adapter pair 1 (NEBNext). Bisulfite conversion was achieved with the Zymo EZ DNA Methylation-Gold kit, following the manufacturer’s instructions. To recover damaged fragments we added 5 μL of 10 mM (5′-CAA GCA GAA GAC GGC ATA CGA GAT TGG TCA GTG ACT GGA GTT CAG ACG TGT GCT CTT CCG ATC T-3′), 200 μM dNTPs, 10 μL VeraSeq Buffer II (Enzymatics) and 1U VeraSeq Ultra (Enzymatics). Following incubation at 95°C for 3 minutes and annealing at 54°C for 45 seconds, extension at 72°C was carried out for 30 minutes. The reaction mixture was incubated with 60 μg of streptavidin coated magnetic beads (Magnasphere Paramagnetic Particles, Promega) in 2x binding buffer (10 mM Tris-HCl pH 7.4, 1 mM EDTA, 2 M NaCl, 0.1% Tween 20) for 20 minutes at room temperature. Beads were washed three times with 400 μL binding buffer before dA-tailing, and a further three times with 400 μL binding buffer before ligation of adapter pair 2. Finally, three washes with 400 μL binding buffer were followed by elution of the non-uracil strand with 50 mM NaOH at 60°C for 15 minutes. The repeats discussed in the manuscript were generated by performing three independent repeats of this protocol on 50 ng samples of sonicated DNA from one biological sample.

### PCR-BS protocol

50 ng of sonicated genomic DNA was end repaired and dA-tailed, before ligation of methylated Illumina sequencing adapters (NEBNext). Bisulfite conversion was achieved with the EZ DNA Methylation-Gold kit (Zymo Research), following the manufacturers instructions. The converted libraries were amplified by PCR, using the VeraSeq Ultra DNA polymerase (Enzymatics). For the *P. berghei* sample 18 cycles were required due to the poor amplification of the AT-rich genome, as previously described for *P. falciparum*[22]. This protocol was performed twice independently on 50 ng samples of sonicated DNA from the same biological sample as used for the ReBuilT protocol.

### Bioinformatic analysis

Detailed information regarding data analysis, and any custom scripts employed, are available in a git repository at github.com/dariober/rebuilt-enhanced-methyl-seq.

### Sequencing and alignment

Raw sequencing files can be found in the GEO under accession number GSE65116 or at http://www.ncbi.nlm.nih.gov/geo/query/acc.cgi?acc=GSE65116. Sequencing was performed on an Illumina NextSeq 500, run in paired-end mode with 75 or 100 read cycles. Reads were trimmed to remove adapter contamination and low quality bases using trim_galore version 0.3.7 and cutadapt version 1.4.2 [28] with option –stringency 3 and default arguments. Trimmed reads were aligned with bwameth.py[29]. The reference sequence was *M. musculus* version mm9 concatenated to *P. berghei* version 11. After alignment, the mapping quality of reads mapped with more then 10% of mismatches was reset to 0 using resetHighMismatchReads.py. Overlapping read pairs were clipped using clipOverlap in the BamUtil suite version 1.0.12. Genomic data manipulations were facilitated by samtools[30], BEDTools[31], Picard and deepTools[32].

### Methylation calling

Counts of converted and unconverted cytosine, i.e: the methylation status, in the *P. berghei* genome were obtained from the alignment files using bam2methylation.py. Only reads with mapping quality greater than 15 were considered, and bases with quality below 13 were also excluded. In addition, at each cytosine position the number of mismatches, i.e: the number of reads not A or C, was recorded. Methylation levels at individual cytosines were assessed independently for each library. At each position a Fisher test was applied to the test if the unconverted cytosines exceeded the number of mismatches found at that position. The p-values from the three ReBuilT and two PCR-BS libraries were combined via Stouffer’s method and corrected for multiple testing [33]. Data analysis was performed in R version 3.1.2 [34].

### Segmenting methylation

Runs of methylated cytosines were detected by segmenting the signal of combined p-values. To this end, the vector of combined p-values was first converted to a vector of discrete observations as follows: ‘0’ if *p* > 0.1, ‘1’ if 0.1 ≤ *p* < 0.05, ‘2’ if 0.05 ≤ *p* < 0.001 and ‘3’ if *p* ≤ 0.001. Then a two state hidden Markov model was fitted to the p-values to partition the signal into segments of high and low methylation. The R package RHmm was used for model fitting [35].

## Supporting Information

S1 Fig The effect of sequence composition on coverage. ReBuilT and PCR-BS libraries were generated from *E. coli* DNA. The normalized read count is plotted against local GC content of the reference genome in 300 base pair windows. Ideally, the GC content of a window should have no impact on the read count. ReBuilT data shows a reduced sensitivity compared to the PCR-BS data.
(EPS)

S2 Fig Genome browser views of *E. coli*. Example regions from *E. coli* sequencing data that are significantly underrepresented through PCR-BS, but are well covered with ReBuilT.
(EPS)

S3 Fig Mass spectrometry analysis of *P. berghei* genomic DNA. A) LC-MS/MS extracted ion counts. The measured transitions consist of the three genomic nucleosides and the spiked in stable isotope labeled standards i) dC, 242 → 112.05054 ii) dC ^15^N_3_, 245 → 115.04164 iii) mC, 242 → 126.06619 iv) mC D_3_, 245 → 129.08502 v) N6MeA, 266 → 150.07742 vi) N6MeA D_3_, 269 → 153.09625 B) Standard curve for 5hmC detection. No 5hmC was detected; limit of detection was 1/10,000 C.
(EPS)

S4 Fig Distribution of insert size in sequenced libraries. Genomic *P. berghei* DNA was sonicated to 250 bp. The sequenced insert size was similar for ReBuilT (mean 111; mode 60) and PCR-BS (mean 108; mode 81). However, while most reads were shorter in the ReBuilT method, there were more reads in the 200–300bp range (8.1% vs 4.36%).
(EPS)

S5 Fig Distribution of read depth across libraries. Libraries were down-sampled to approximately 29 million reads to be comparable with each other. The read count was computed in 50 base pair windows across the *P. berghei* genome, normalized to Reads Per Million (RPM), and plotted for each library. The ReBuilT libraries exhibit a higher median value and a lower standard deviation that the PCR-BS libraries. (ReBuilT: 2.7 ± 1.7; PCR-BS: 1.9 ± 5.1).
(EPS)

S6 Fig Duplication rates. Duplicate reads obtained using read one only. The dashed horizontal line indicates the expected duplication rate given the read number and genome size. The ReBuilT libraries show a small increase over the expected value, while the PCR-BS libraries show over double the expected duplication rate. The observed duplication rate includes PCR duplicates, but is also affected by uneven coverage.
(EPS)

S7 Fig Effect of sequence composition on coverage for individual libraries. The normalized read count is plotted against local GC content of the reference genome in 300 base pair windows. Ideally, the GC content of a window should have no impact on the read count. The ReBuilT libraries exhibit little sensitivity to the percent GC, while the PCR-BS libraries exhibit a strong preference for more balanced base compositions.
(PDF)

S8 Fig Methylation is detected at sites with poor coverage. Increasing read counts should increase the quantitative power of bisulfite, i.e: lower methylation levels can be detected. Conversely, the quantitative power of bisulfite sequencing is low where the read counts are low. (Top) As ReBuilT exhibits even coverage, all regions have sufficient read depth for accurate methylation calling. (Bottom) Due to the uneven coverage of PCR-BS, many regions have low read counts, and in these regions the observed percent methylation is suspiciously high.
(EPS)

S9 Fig Characteristics of methylated segments in *P. berghei*. a) Methylated segments containing greater than 5 cytosines, as detected by a hidden Markov model. Left, the distribution of segment length. Right, the distribution of average percent methylation. b) An example of a methylated segment; each bar represents a cytosine along the plotted region. The top panel shows the percentage methylation, and the bottom panel shows the -log10 of the p-value for the presence of methylation.
(EPS)

S10 Fig Comparison of C to T conversion measured with the PCR-BS and ReBuilT methods. The bisulfite induced C to T conversion of bisulfite-treated E14 mouse embryonic stem cells was analysed with the PCR-BS and ReBuilT protocols. The conversion rates are similar between the two methods, though the PCR-BS rates are generally higher as seen by the skew below the diagnol. This is likely due to PCR amplification artefacts.
(EPS)

S1 Protocol A detailed ReBuilT protocol. A step-by-step protocol for the ReBuilT methodology.
(PDF)

S1 Scheme ReBuilT scheme with sequences. Adapter pair 1 comprises a full length Illumina adapter modified with a 3′ biotin, and a short complementary sequence blocked with a 3′ dideoxythymidine (ddT). The ddT aids ligation efficiency and specificity, yet is not ligated due to the lack of a 3′ hydroxyl. All cytosines in the full length partner of adapter pair one are 5mCs to protect against deamination during bisulfite treatment. Adapter pair 2 comprises a fully complementary Illumina adapter sequence, with a 3′ T overhand to aid ligation.
(EPS)

S1 Table ReBuilT rescues damaged fragments to increase the concentration of sequenceable fragments. Sequencing libraries were prepared from *E. coli* genomic DNA with both the ReBuilT method and a standard BS-seq protocol. The concentrations of sequenceable fragments in the libraries were two orders of magnitude higher for ReBuilT that the traditional protocol pre-PCR. The earlier the threshold cycle (Ct) is reached, the more DNA was initially present.
(PDF)

S2 Table Mass spectrometry data. Quantitative mass spectrometry was performed on the biological sample used to generate sequencing libraries. Concentrations for dC and mC were back calculated from a calibration curve using standard solutions for all nucleosides. Reported values for percent mC are calculated from total C.
(PDF)

S3 Table Sequencing quality. The mode of the average sequence quality, and the associated percentage, of all reads. The ReBuilT libraries exhibit higher modal Phred scores than the PCR-BS libraries.
(PDF)

S4 Table *P. berghei* methylation. A cytosine base was called as a methylated loci if the FDR corrected P-value was less than 0.01, and the percentage methylation at a site was calculated as *C*/(*C* + *T*).
(PDF)

S5 Table Methylation in E14 mouse embryonic stem cells by context. The methylation status of cytosines in different nucleotide contexts was investigated for E14 mouse embryonic stem cells. This table shows the results for chromosome 19 only.
(PDF)

S6 Table List of oligonucleotides. The custom oligonucleotides employed in the ReBuilT protocol. 5-methylcytosine is represented by a 5.
(PDF)
