# Supplementary figures and images for: Enhanced Methylation Analysis by Recovery of Unsequenceable Fragments
Source: PLoS One. 2016 Mar 31;11(3):e0152322. doi: 10.1371/journal.pone.0152322 (PMC4816320; doi:10.1371/journal.pone.0152322)

REBUiLT\_1

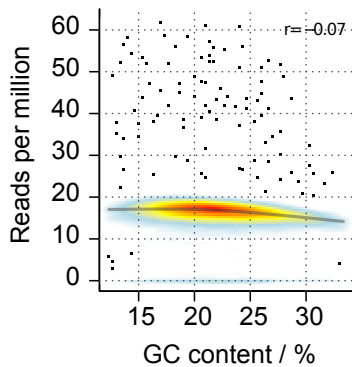

REBUiLT\_2

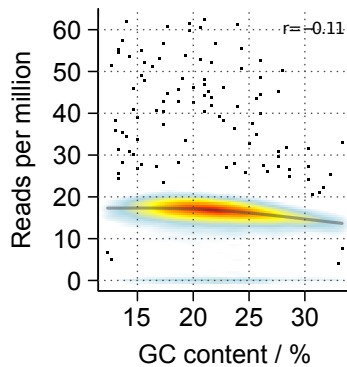

REBUiLT\_3

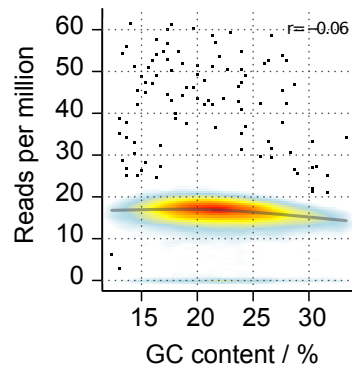

PCR-BS\_1

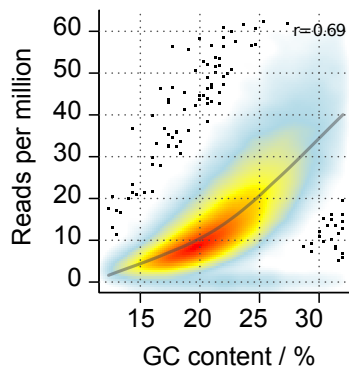

PCR-BS\_2

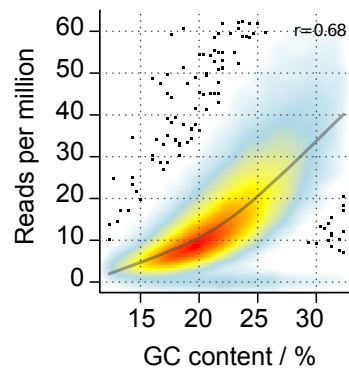

Supplement: S7 Fig — The normalized read count is plotted against local GC content of the reference genome in 300 base pair windows. Ideally, the GC content of a window should have no impact on the read count. The ReBuilT libraries exhibit little sensitivity to the percent GC, while the PCR-BS libraries exhibit a strong preference for more balanced base compositions. (PDF) [file pone.0152322.s007.pdf]
