# Supplementary material for: Enhanced Methylation Analysis by Recovery of Unsequenceable Fragments
Source: PLoS One. 2016 Mar 31;11(3):e0152322. doi: 10.1371/journal.pone.0152322 (PMC4816320; doi:10.1371/journal.pone.0152322)
Supplement: S1 Protocol — A step-by-step protocol for the ReBuilT methodology. (PDF) [file pone.0152322.s011.pdf]

Enhanced methylation analysis of Plasmodium berghei by recovery of unsequenceable fragments

*Gordon R. McInroy, Dario Beraldi, Eun-Ang Raiber, Katarzyna Modrzynska, Pieter van Delft, Oliver Billker, Shankar Balasubramanian*

## Detailed Experimental Protocol

1) DNA fragmentation

***Covaris M220 sonication***
Sonicate genomic DNA in 50uL 1X TE buffer (10mM TRIS-HCl pH 8.0, 0.1 mM EDTA) to an appropriate fragment size. E.g.: for 250 bp peak use the following settings: incident power 75 W, duty factor 10%, cycles per burst 200, treatment time 130s.

2) End Repair (NEB Next Kit)

| | | |
|---|---|---|
| DNA | 1ug | (in 85uL water or 10mM Tris-HCl pH7.4) |
| End repair buffer | 10uL | |
| End repair enzyme | 5uL | |
| Total | 100uL | |

- *Mix by gently pipetting entire volume up and down 10x*
- *Incubate for 30min at 20°C*
- *Purification (column or ampure beads)*
- *Elution in 44uL water*

3) A-tailing (NEB Next Kit)

| | |
|---|---|
| DNA | 42uL |
| Buffer | 5uL |
| Enzyme | 3uL |
| Total | 50uL |

- *Mix by gently pipetting entire volume up and down 10x*
- *Incubate for 30min at 37°C*
- *Purification (column or ampure beads)*
- *Elution in 35uL water*

4) Ligation (Next Next Kit)

| | |
|---|---|
| DNA | 32.5uL |
| Ligation buffer | 10uL |
| Adapter pair A | 2.5uL |
| Ligase | 5uL |
| Total | 50uL |

- *Incubate for 20min at 20°C (r.t. is not 20°C)*
- *Add 1.6x ampure beads for removal of small DNA fragments*
- *Wash beads*
- *Elute with 22uL water, recover 20uL (max for oxBS workflow)*

5) Enter BS protocol

***Perform bisulfite conversion protocol of choice, following manufacturer instructions. Once converted DNA is purified, proceed to step 6). This protocol has been established using the EZ DNA Methylation-Gold Kit from Zymo Research.***

6) Single extension (eg: with VeraSeq ultra or KAPA HiFi Uracil +)

| | | |
|---|---|---|
| DNA | 20uL | |
| LM primer | 5uL | (10uM stock concentration) |
| dNTP | 1uL | (10mM stock concentration) |
| Buffer | 10uL | |
| Veraseq Ultra | 0.5uL | |
| Water | 13.5uL | |
| Total | 50uL | |

| | |
|---|---|
| *Denaturation* | *95°C for 3 min* |
| *Annealing* | *54°C for 45 sec* |
| *Extension* | *74°C for 30 min* |

- *Purification with 60uL streptavidin coated magnetic beads (Promega Magnasphere; 1 mg/mL)*
  - *Wash 60uL beads with 1X BB (4x 300uL)*
    - *10 mM Tris pH 7.5*
    - *1 mM EDTA*
    - *2M NaCl*
    - *0.1% Tween-20*
  - *Resuspend beads in 50uL 2X BB*
  - *Add 50uL reaction mixture and incubate for 20 minutes at room temperature*
  - *Wash beads with 1X BB (3x 300uL)*

7) End repair of singly adapted library

| | |
|---|---|
| End repair buffer | 10uL |
| End repair enzyme | 5uL |
| Water | 85uL |
| Total | 50uL |

- *Incubate for 30min at 20°C*
- *Wash beads with 1X binding buffer (3x400uL)*

8) A-tailing

| | |
|---|---|
| Buffer | 5uL |
| Enzyme | 3uL |
| Water | 42uL |
| Total | 50uL |

- *Incubate for 30min at 37°C*
- *Wash with 1X binding buffer (3x400uL)*

9) Ligation of adapter pair B (NEB Next Quick Ligation Module)

- *Oligos 1b and 2b must be annealed to a final concentration of 25uM in 10mM Tris-HCl pH 7.4 and 50mM NaCl (annealing conditions as in Appendix 1b)*

| | |
|---|---|
| Ligation buffer | 10uL |
| Adapter pair B | 2.5uL |
| Ligase | 5uL |
| Water | 32.5uL |
| Total | 50uL |

- *Incubate for 20min at 20°C*
- *Wash with 1X BB (3x 300uL)*
- *Elution with 20uL NaOH (50mM, fresh dilution from 1M stock), 65°C for 15min*

10) qPCR with Kapa Master Mix

Quantify the library by qPCR with the KAPA quantification kit before sequencing.

Appendix: Annealing conditions for adapters:

Perform the following thermocycler program, with ramp rate at minimum (generally 0.1 °C/s)

| | |
|---|---|
| 95 °C | 15 min |
| 90 °C | 3 min |
| 85 °C | 3 min |
| 80 °C | 3 min |
| 70 °C | 5 min |
| 10 °C | 10 min |
| 4 °C | hold |
