# Supplementary material for: Enhanced Methylation Analysis by Recovery of Unsequenceable Fragments
Source: PLoS One. 2016 Mar 31;11(3):e0152322. doi: 10.1371/journal.pone.0152322 (PMC4816320; doi:10.1371/journal.pone.0152322)
Supplement: S1 Table — Sequencing libraries were prepared from E. coli genomic DNA with both the ReBuilT method and a standard BS-seq protocol. The concentrations of sequenceable fragments in the libraries were two orders of magnitude higher for ReBuilT that the traditional protocol pre-PCR. The earlier the threshold cycle (Ct) is reached, the more DNA was initially present. (PDF) [file pone.0152322.s013.pdf]

		C_t value	
**Protocol**	**Replicate 1**	**Replicate 2**	**Average**
ReBuilT	13.47	13.53	13.50
PCR-BS	20.06	20.21	20.14
