# Supplementary material for: Enhanced Methylation Analysis by Recovery of Unsequenceable Fragments
Source: PLoS One. 2016 Mar 31;11(3):e0152322. doi: 10.1371/journal.pone.0152322 (PMC4816320; doi:10.1371/journal.pone.0152322)
Supplement: S2 Table — Quantitative mass spectrometry was performed on the biological sample used to generate sequencing libraries. Concentrations for dC and mC were back calculated from a calibration curve using standard solutions for all nucleosides. Reported values for percent mC are calculated from total C. (PDF) [file pone.0152322.s014.pdf]

Conc. / nM		Relative composition
**C**	**5mC**	**5mC/(C+5mC)**
11958.256	36.745	3.06×10^{-3}
