# Supplementary material for: Enhanced Methylation Analysis by Recovery of Unsequenceable Fragments
Source: PLoS One. 2016 Mar 31;11(3):e0152322. doi: 10.1371/journal.pone.0152322 (PMC4816320; doi:10.1371/journal.pone.0152322)
Supplement: S3 Table — The mode of the average sequence quality, and the associated percentage, of all reads. The ReBuilT libraries exhibit higher modal Phred scores than the PCR-BS libraries. (PDF) [file pone.0152322.s015.pdf]

**Library**	**Mate**	**Phred mode**	**% reads**	**Protocol**
grm034_ReBuilT_AD04	R1	35	19.33	ReBuilT
grm034_ReBuilT_AD04	R2	34	13.91	ReBuilT
grm035_ReBuilT_AD06	R1	35	21.62	ReBuilT
grm035_ReBuilT_AD06	R2	34	15.17	ReBuilT
grm036_ReBuilT_AD12	R1	35	22.29	ReBuilT
grm036_ReBuilT_AD12	R2	34	15.34	ReBuilT
grm037_PCRBS_AD04	R1	31	13.77	PCRBS
grm037_PCRBS_AD04	R2	32	9.56	PCRBS
grm038_PCRBS_AD16	R1	31	13.94	PCRBS
grm038_PCRBS_AD16	R2	32	10.29	PCRBS
