# Supplementary material for: Enhanced Methylation Analysis by Recovery of Unsequenceable Fragments
Source: PLoS One. 2016 Mar 31;11(3):e0152322. doi: 10.1371/journal.pone.0152322 (PMC4816320; doi:10.1371/journal.pone.0152322)
Supplement: S4 Table — A cytosine base was called as a methylated loci if the FDR corrected P-value was less than 0.01, and the percentage methylation at a site was calculated as C/(C + T). (PDF) [file pone.0152322.s016.pdf]

Protocol	No. methylated loci	Global methylation / %
ReBuilT	76,205	0.69
PCR-BS	327,904	5.91
