# Supplementary material for: Enhanced Methylation Analysis by Recovery of Unsequenceable Fragments
Source: PLoS One. 2016 Mar 31;11(3):e0152322. doi: 10.1371/journal.pone.0152322 (PMC4816320; doi:10.1371/journal.pone.0152322)
Supplement: S5 Table — The methylation status of cytosines in different nucleotide contexts was investigated for E14 mouse embryonic stem cells. This table shows the results for chromosome 19 only. (PDF) [file pone.0152322.s017.pdf]

Method	Context	C	T	non-CT	% met	% non-CT
ReBuilT	CG	1721708	2864698	17164	60.10	0.37
	CHG	90355	14287737	38273	0.63	0.27
	CHH	178905	40077113	147281	0.45	0.36
PCR-BS	CG	5668591	8355691	30318	67.84	0.22
	CHG	317727	40248613	50873	0.79	0.13
	CHH	715017	118302818	140713	0.60	0.12
