# Supplementary material for: Enhanced Methylation Analysis by Recovery of Unsequenceable Fragments
Source: PLoS One. 2016 Mar 31;11(3):e0152322. doi: 10.1371/journal.pone.0152322 (PMC4816320; doi:10.1371/journal.pone.0152322)
Supplement: S6 Table — The custom oligonucleotides employed in the ReBuilT protocol. 5-methylcytosine is represented by a 5. (PDF) [file pone.0152322.s018.pdf]

**Oligomer**	**Sequence (5′ to 3′)**
ODN1a	GCT CTT CCG ATC (ddT)
ODN1b	GAT 5GG AAG AG5 A5A 5GT 5TG AA5 T55 AGT 5AC TGA 55A AT5 T5G TAT G55 GT5 TT5 TG5 TTG-(biotin)
ODN2a	AAT GAT ACG GCG ACC ACC GAG ATC TAC ACT CTT TCC CTA CAC GAC GCT CTT CCG ATC T
ODN2b	GAT CGG AAG AGC GTC GTG TAG GGA AAG AGT GTA GAT CTC GGT GGT CGC CGT ATC ATT
ODN3	CAA GCA GAA GAC GGC ATA CGA GAT TGG TCA GTG ACT GGA GTT CAG ACG TGT GCT CTT CCG ATC T
